# Editorial: Community series in ICD-11 personality disorders: utility and implications of the new model, volume II

**DOI:** 10.3389/fpsyt.2023.1199703

**Published:** 2023-05-22

**Authors:** Antonella Somma, Jared W. Keeley, Bo Bach

**Affiliations:** ^1^School of Psychology, Vita-Salute San Raffaele University, Milan, Italy; ^2^Clinical Psychology and Psychotherapy Unit, IRCCS San Raffaele Turro Hospital, Milan, Italy; ^3^Psychology Department, Virginia Commonwealth University, Richmond, VA, United States; ^4^Center for Personality Disorder Research (CPDR), Psychiatric Research Unit, Slagelse Psychiatric Hospital, Slagelse, Denmark

**Keywords:** ICD-11, personality disorder, diagnosis, clinical utility, severity of personality disorder, trait domains, cross-cultural

The 11th edition of the *International Classification of Diseases* [(ICD-11); (1)] was approved in 2019 for countries to start adopting in January 2022. The ICD-11 proposed a significant shift in the assessment of personality disorders (PDs), moving toward a more evidence-based approach to the classification of personality dysfunction. Indeed, a new dimensional approach to the diagnosis of PD was introduced, and clinicians are now required to think about their patients and clients in terms of the severity of their core personality dysfunction, relying on a global evaluation of self- and interpersonal functioning along with emotional, cognitive, and behavioral manifestations (1). After establishing if the person manifests a PD, the practitioner may determine whether the patient's level of personality problems corresponds to a Mild, Moderate, or Severe PD. Notably, to characterize the specific traits (i.e., style) defining the global impairment, clinicians may model a personality profile relying on five broad personality domains (i.e., Negative Affectivity, Detachment, Dissociality, Disinhibition, and Anankastia). Finally, the ICD-11 PD model includes a Borderline Pattern specifier to facilitate access to existing evidence-based treatments.

Given the interest in the new model (2, 3), we build on the first volume of this Research Topic to provide readers with new insights into the ICD-11 PD model. All the studies included in this second volume are concerned with a common theme: Providing evidence to support the clinical usefulness of the model from different perspectives. Indeed, the ICD-11 PD model, as a diagnostic system, aims at providing clinicians with diagnostic guidance that is perceived as useful for clinical practice (3), by practitioners working with various treatment models (4), and across different cultural contexts (5). As a whole, the papers included in this second volume deal with these issues, while representing an attempt at addressing some areas of potential development of the ICD-11 PD model, hopefully promoting reflection on its possible future advancement. In the following paragraphs, we will try to point out key findings of the articles, focusing on three main characteristics of the ICD-11 PD model: (a) relationships with other dimensional models of personality pathology, (b) usefulness for existing treatment models, and (c) a cross-cultural perspective.

## Relationships with other dimensional models of personality pathology

Dimensional models of PDs have been shown to not only be empirically sound (6), but also to be more useful from a clinical standpoint (7, 8). Thus, not surprisingly, both the latest edition of the Diagnostic and Statistical Manual of Mental Disorders [(DSM-5); (9)] in Section III and ICD-11 have moved toward dimensional models of personality pathology. To diagnose a PD, the two diagnostic systems propose to focus on core impairment in self and interpersonal functioning and on four similar trait domains (the only exception being the ICD-11 domain of Anankastia, which is not directly identified in the DSM-5, and the DSM-5 Psychoticism domain that is not included in ICD-11). Against this background, examining the overlap and distinctions between the ICD-11 and DSM-5 models is relevant (10), and may pave the ground for future studies exploring their potential usefulness in treatment planning and monitoring (e.g., the relevance of Psychoticism and/or Anankastia for therapy outcomes). Starting from the recognition of the differences in selected DSM-5 and ICD-11 domains, Bastiaens et al. investigated the utility of a combined ICD-11/DSM-5 trait framework to describe personality problems, while considering the role played by identity, a central aspect in the definition of personality dysfunction. Interconnections between impairment in personality functioning and trait domain expression are expected, and their magnitude represents a challenge for both ICD-11 and DSM-5 PD models (11). Two studies in this volume examined this issue. Pires et al., assessed the potential usefulness of the PID5BF+M in capturing personality dysfunctions, whereas Gutiérrez et al. focused on the strong interconnections between the severity of personality dysfunction and the five personality domains and the borderline specifier, and proposed some possible insights for future model refinement.

## Usefulness for existing treatment models

Notably, one of the ambitions of the ICD-11 PD model is to strengthen the link between diagnosis and treatment planning, allowing clinicians to strongly rely upon information collected during the diagnostic assessment to manage treatment (12). From this perspective, it is particularly important to consider the potential usefulness of the ICD-11 PD model for different treatment strategies and therapeutic approaches. For instance, Tracy et al. (4) showed the clinical utility of the ICD-11 for guiding treatment decision-making focusing on schema therapy and dialectical behavior therapy, while Sharp and Bevington (13) suggested that focusing on the ICD-11 severity criterion may be useful in order to express the diagnosis in relational terms, which is consistent with Mentalization Based Treatment (14). As Blüml and Doering (15) nicely described in a paper included in the first volume of this collection, there is significant overlap between ICD-11 description of personality functioning and psychoanalytic conceptualizations of personality. Notably, Bach and Simonsen (16) build on the evidence that ICD-11 PD severity conceptualization is based on the same core capacities of self and interpersonal functioning postulated by Kernberg's Levels of Personality Organization and proposed a “cross walk” for level of personality functioning with respect to sense of self. In this line of research, the study presented in this volume by Unoka et al. provided empirical support to the crosswalk proposed by Bach and Simonsen (16). As nicely reported by Bach and First (17), the ICD-11 domain specifiers allow clinicians to focus their attention on the person's unique trait domain profile, providing clinical information for selecting the most adequate therapeutic strategy based on the required focus (12). Riegel et al. study focused on antagonist/dissocial features (i.e., narcissistic personality characteristics), and provided empirical support for the clinical utility of the new model among individuals with addiction problems.

## Cross-cultural perspective

Interestingly, five out of the six studies included in this second volume presents data collected from diverse European countries (i.e., Belgium, Czech Republic, Portugal, Spain, and Hungary). This is not surprising because European countries have often been the first to migrate to a new ICD classification (18). At the same time, cultural aspects are crucial for the possibility of applying the ICD-11 PD model across continents (19). Since the release of the new model, significant research efforts have been made to test the cross-cultural sustainability of the system (3, 6), showing its potential usefulness. The paper by Hualparuca-Olivera takes another step further, and directly examined the role of culture in the implementation of the ICD-11 model, examining the opportunities and challenges offered by the new PD system through the lenses of Peruvian social structure, geographical differences, and cultural perceptions of personality.

## Future directions

Research on the ICD-11 PD model is accumulating, and results seem to be promising. Of course, additional research connecting ICD-11 PD model features to specific treatment techniques is needed. However, the possibility to empirically test the clinical usefulness of the model across different healthcare settings across the world rely on clinicians adopting the new system to assess and describe personality disturbances of their clients, and we hope that the papers presented in this volume would encourage new efforts in this direction.

## Author contributions

AS wrote the first draft of the manuscript. All authors contributed to manuscript revision, read, and approved the submitted version.
